# Supplementary material for: Genome-scale functional mapping of the mammalian whole brain with in vivo Perturb-seq
Source: bioRxiv. 2026 Mar 18:2026.03.16.711480. Preprint. [Version 1] doi: 10.64898/2026.03.16.711480 (PMC13108593; doi:10.64898/2026.03.16.711480)
Supplement: 1 [file NIHPP2026.03.16.711480V1-supplement-1.pdf]

bioRxiv preprint doi: https://doi.org/10.64898/2026.03.16.711480; this version posted March 18, 2026. The copyright holder for this preprint (which was not certified by peer review) is the author/funder, who has granted bioRxiv a license to display the preprint in perpetuity. It is made available under aCC-BY 4.0 International license.

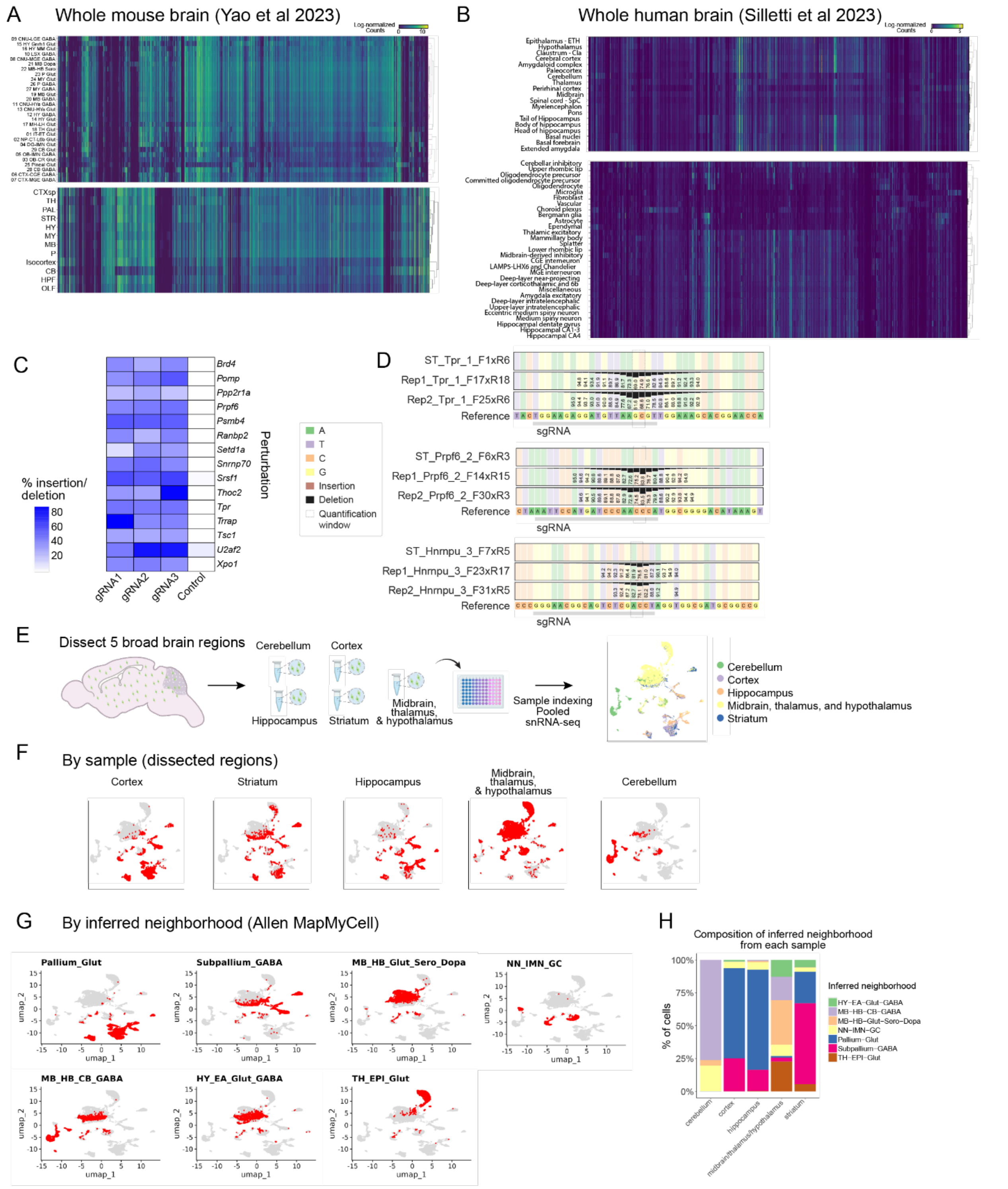

Figure S1. Expression pattern of 1,947 risk genes, in vitro gRNA activity test, and MapMyCells fidelity test. (A) Heatmap showing the gene expression levels of 1,947 neurodevelopmental disease-associated risk genes perturbed in this study across different cell types using whole mouse brain atlas[6]. **(B)** Heatmap showing the gene expression levels of 1,947 neurodevelopmental disease-associated risk genes

bioRxiv preprint doi: https://doi.org/10.64898/2026.03.16.711480; this version posted March 18, 2026. The copyright holder for this preprint (which was not certified by peer review) is the author/funder, who has granted bioRxiv a license to display the preprint in perpetuity. It is made available under aCC-BY 4.0 International license.

perturbed in this study across different cell types using adult human brain atlas[2]. **(C)** Heatmap of in vitro gRNA activity of 45 selected gRNAs (15 genes, 3 gRNAs per gene) compared to safe-targeting controls by insertion-deletion analysis. **(D)** Examples of nucleotide composition and indel frequency near gRNA cut sites in selected guides (Tpr_1, Prpf6_2, Hnrnpu_3). **(E)** Schematic of snRNA-seq experiment profiling separate brain regions to test MapMyCell[86] cell type assignment fidelity. **(F-G)** UMAPs of a single-nucleus Flex RNA-seq library collected from whole mouse brains with each major brain region dissected and nuclei extracted separately. Each dissection region (F) and inferred neighborhood (G) are highlighted. **(H)** Stacked bar plot showing percentage of cells from inferred neighborhood recovered from each dissection region.

bioRxiv preprint doi: https://doi.org/10.64898/2026.03.16.711480; this version posted March 18, 2026. The copyright holder for this preprint (which was not certified by peer review) is the author/funder, who has granted bioRxiv a license to display the preprint in perpetuity. It is made available under aCC-BY 4.0 International license.

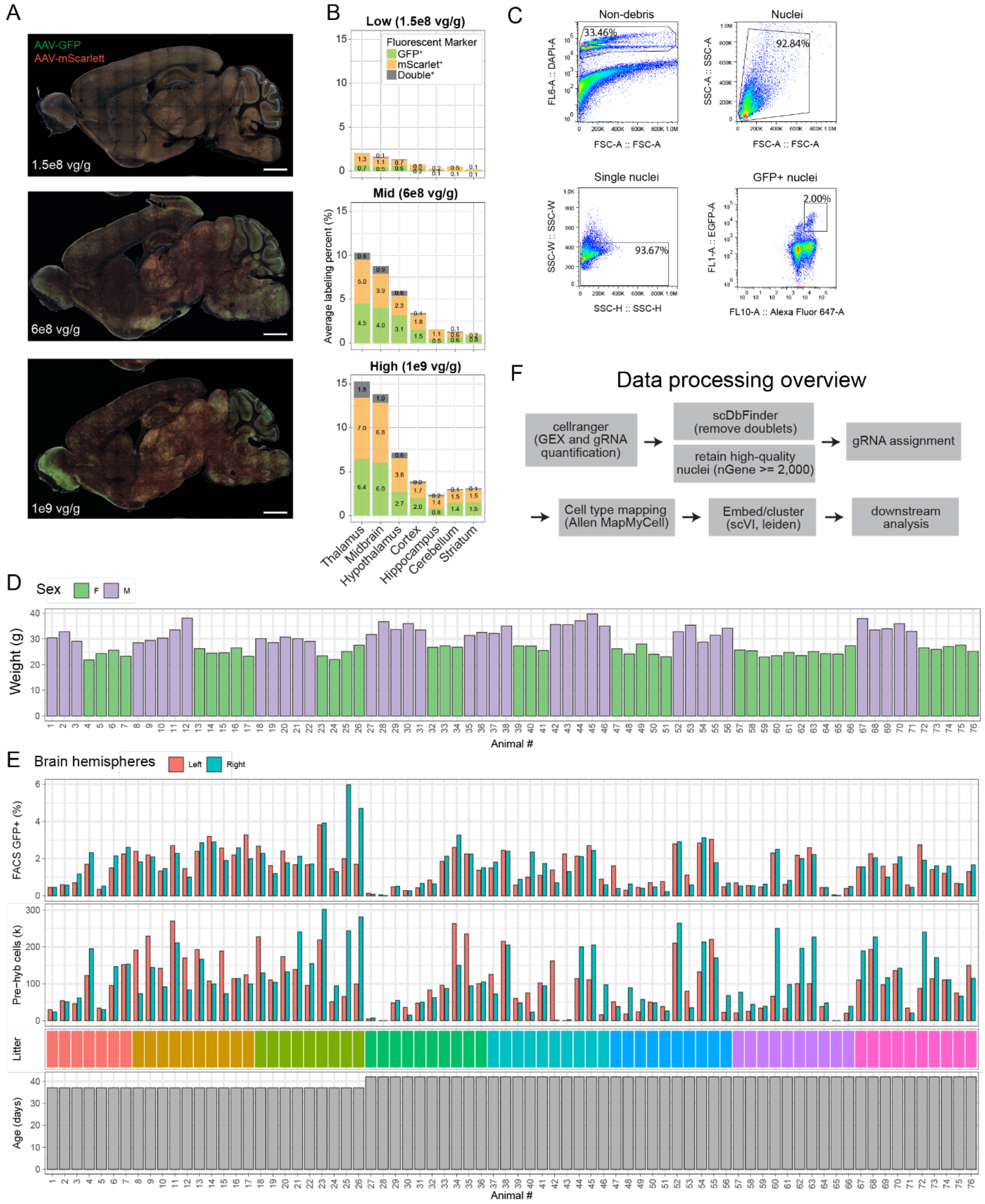

Figure S2. in vivo AAV titer optimization, snRNA-seq data processing, and animal-level metadata. (A) Immunofluorescence image of sagittal section of a P37 mouse brain retro-orbitally injected at P16 with high (1e9), mid (6e8), or low (1.5e8) total vg per gram of body weight of AAV PHP.eB encoding either GFP or mScarlet (1:1 ratio) (scale bar = 1 mm). **(B)** Quantification of GFP and mScarlet viral labeling efficiency as

bioRxiv preprint doi: https://doi.org/10.64898/2026.03.16.711480; this version posted March 18, 2026. The copyright holder for this preprint (which was not certified by peer review) is the author/funder, who has granted bioRxiv a license to display the preprint in perpetuity. It is made available under aCC-BY 4.0 International license.

well as double labeling rate in (A). **(C)** Representative FACS gating strategy to enrich transduced neuronal nuclei. **(D)** Bar plot of sex and weight at harvest of animals used in this study. **(E)** Animal tracking information showing the litter, age at harvest for each animal, as well as AAV-labeling rate by FACS and total nuclei number per hemisphere used for Flex hybridization. **(F)** Schematic of snRNA-seq data processing and quality control workflow.

bioRxiv preprint doi: https://doi.org/10.64898/2026.03.16.711480; this version posted March 18, 2026. The copyright holder for this preprint (which was not certified by peer review) is the author/funder, who has granted bioRxiv a license to display the preprint in perpetuity. It is made available under aCC-BY 4.0 International license.

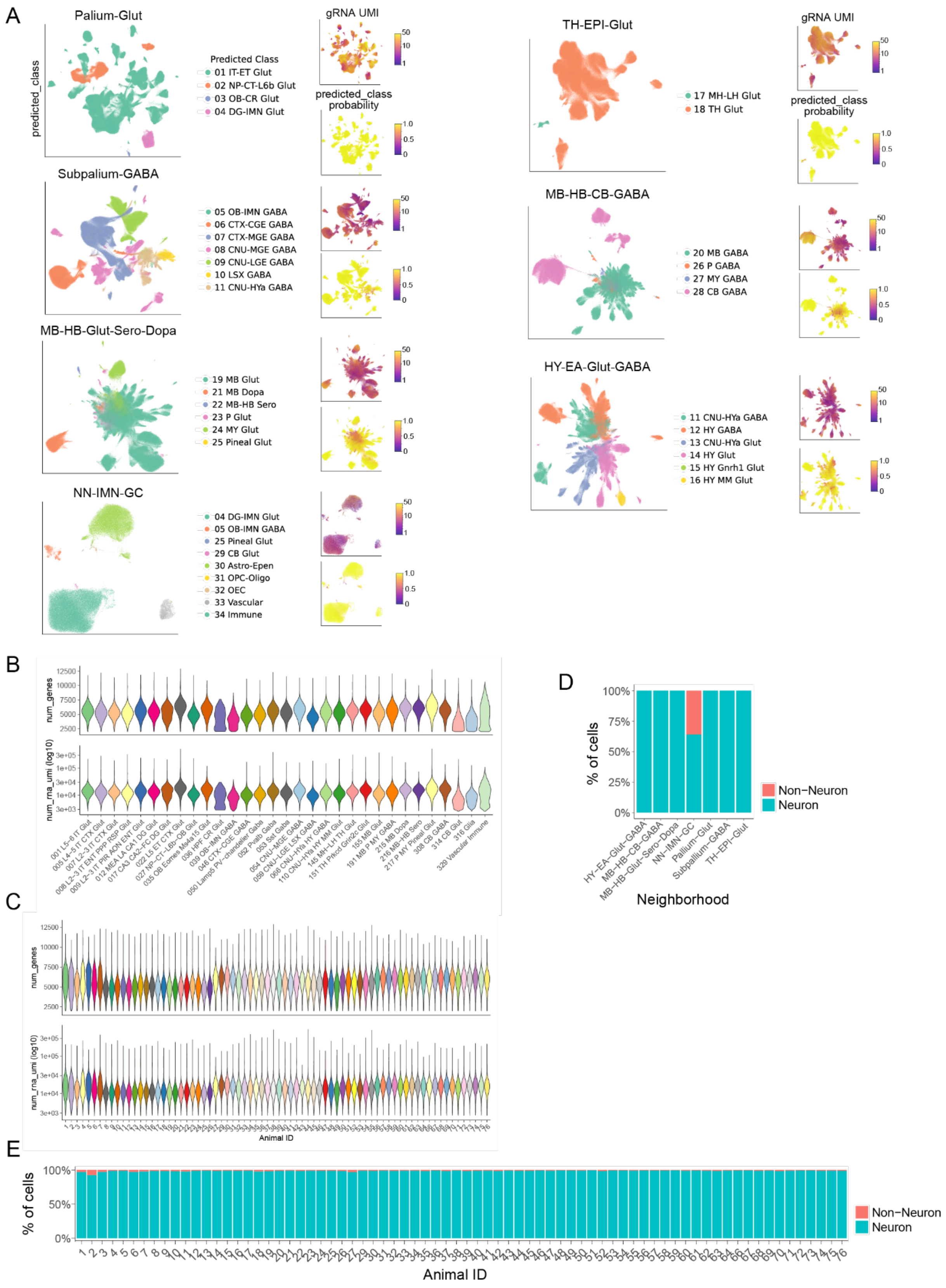

Figure S3. Additional visualization and quality control of snRNA-seq data. (A) UMAPs of whole brain in vivo Perturb-seq dataset separated by neighborhoods, colored

bioRxiv preprint doi: https://doi.org/10.64898/2026.03.16.711480; this version posted March 18, 2026. The copyright holder for this preprint (which was not certified by peer review) is the author/funder, who has granted bioRxiv a license to display the preprint in perpetuity. It is made available under aCC-BY 4.0 International license.

by inferred cell class and predicted class probability using MapMyCells[86], as well as number gRNA UMIs per nucleus. **(B)** Violin plots of the number of genes and RNA UMIs recovered per nucleus from each binned cell type. **(C)** Violin plots of the number of genes and RNA UMIs recovered per nucleus from each animal. **(D)** Bar plot of % neuronal versus non-neuronal population recovered from each developmental neighborhood. **(E)** Bar plots of % neuronal versus non-neuronal population recovered from each animal.

bioRxiv preprint doi: https://doi.org/10.64898/2026.03.16.711480; this version posted March 18, 2026. The copyright holder for this preprint (which was not certified by peer review) is the author/funder, who has granted bioRxiv a license to display the preprint in perpetuity. It is made available under aCC-BY 4.0 International license.

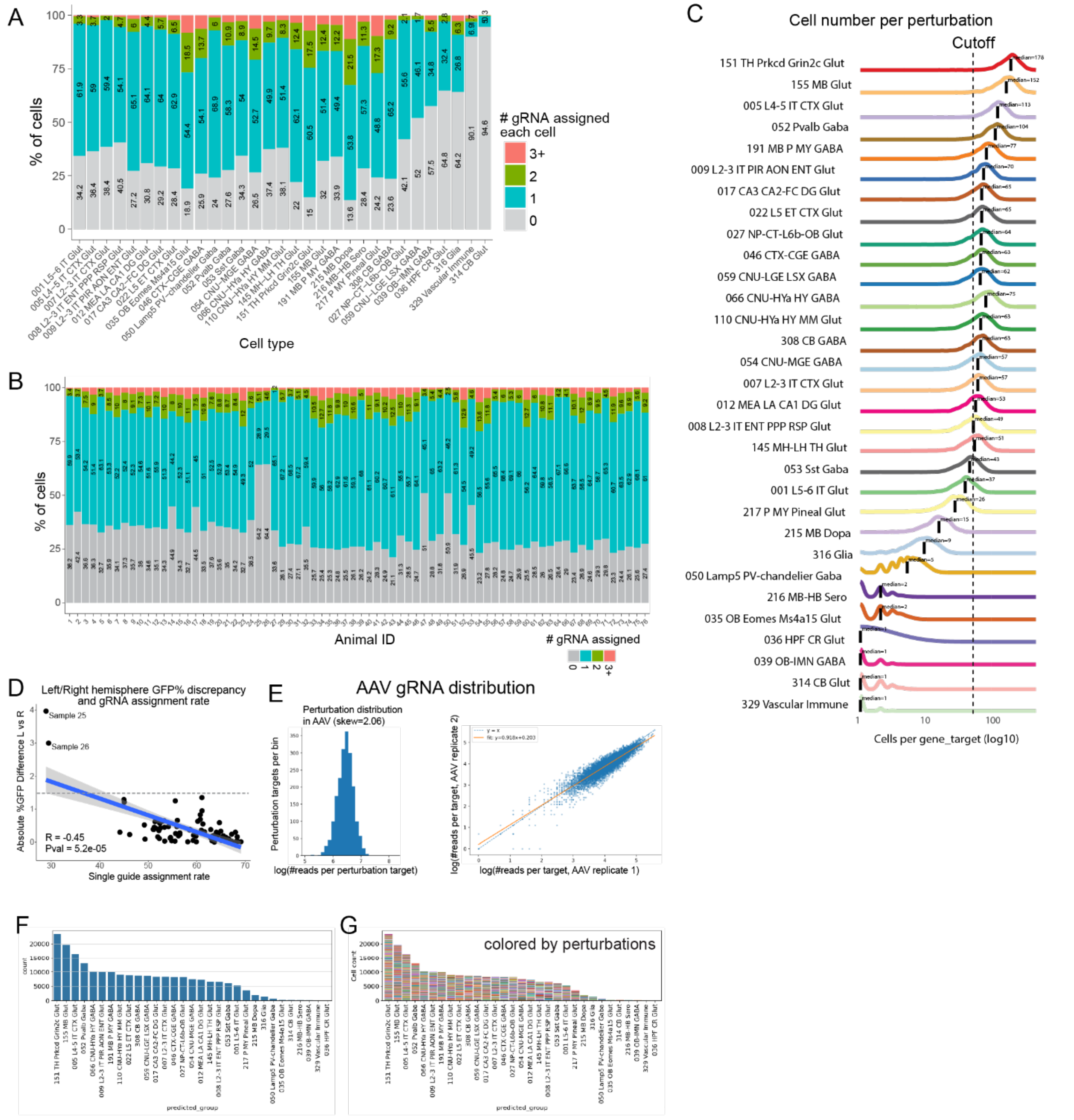

Figure S4. gRNA and gRNA assignment quality control. (A-B) Stacked bar plots of percentage of nuclei with no guide, single, double, or multiple guide assignment from each binned cell type (A) and animal (B). **(C)** Density curves of nuclei number recovered per perturbation separated by cell types. Vertical dashed line indicates minimum number of nuclei per perturbation cutoff for downstream analyses. **(D)** Scatterplot showing single gRNA assignment correlation to discrepancy for FACS labeling rate between left and right hemispheres within one animal (discrepancy in FACS gating between samples). **(E)** Histogram of 8,588 gRNA distribution in AAV library and scatter plot of gRNA correlation between two different batches of viral preparation. **(F)** Ranked bar plot of total number of nuclei assigned with single perturbation identity for each

bioRxiv preprint doi: https://doi.org/10.64898/2026.03.16.711480; this version posted March 18, 2026. The copyright holder for this preprint (which was not certified by peer review) is the author/funder, who has granted bioRxiv a license to display the preprint in perpetuity. It is made available under aCC-BY 4.0 International license.

binned cell type. **(G)** Ranked bar plot of total number of nuclei assigned with single perturbation identity for each binned cell type, colored by perturbation identity.

bioRxiv preprint doi: https://doi.org/10.64898/2026.03.16.711480; this version posted March 18, 2026. The copyright holder for this preprint (which was not certified by peer review) is the author/funder, who has granted bioRxiv a license to display the preprint in perpetuity. It is made available under aCC-BY 4.0 International license.

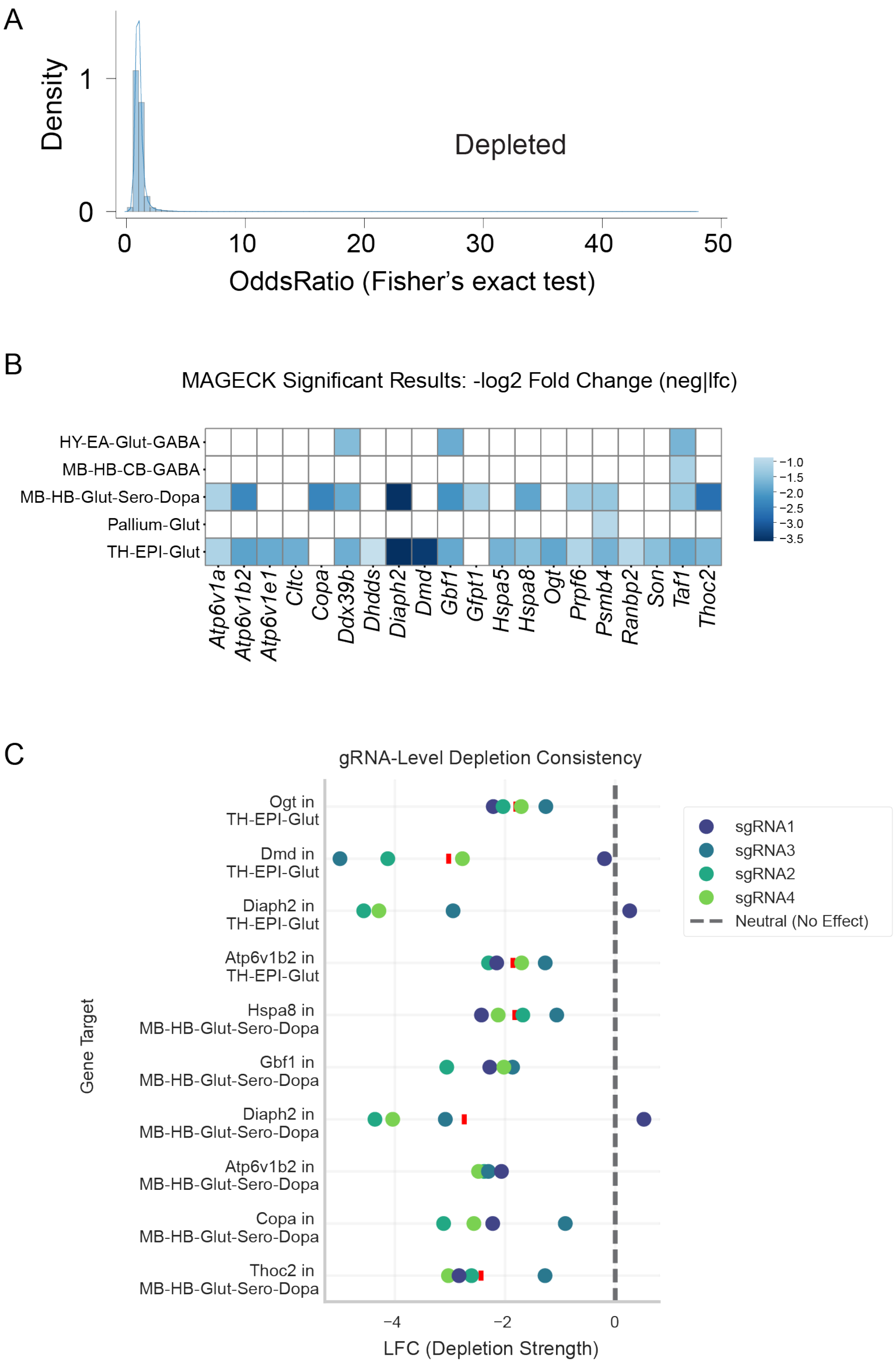

Figure S5. Cell type depletion analysis with Fisher's exact test and MAGeCK. (A) Density plot of odds ratios for all depleted perturbation–cell type pairs using Fisher's exact test. **(B-C)** MAGeCK significant results ($\log_2$ fold-change) for depleted perturbation–cell type pairs across binned neuronal classes, concordant with Fisher's exact test results shown in Fig. 2A-C.

bioRxiv preprint doi: https://doi.org/10.64898/2026.03.16.711480; this version posted March 18, 2026. The copyright holder for this preprint (which was not certified by peer review) is the author/funder, who has granted bioRxiv a license to display the preprint in perpetuity. It is made available under aCC-BY 4.0 International license.

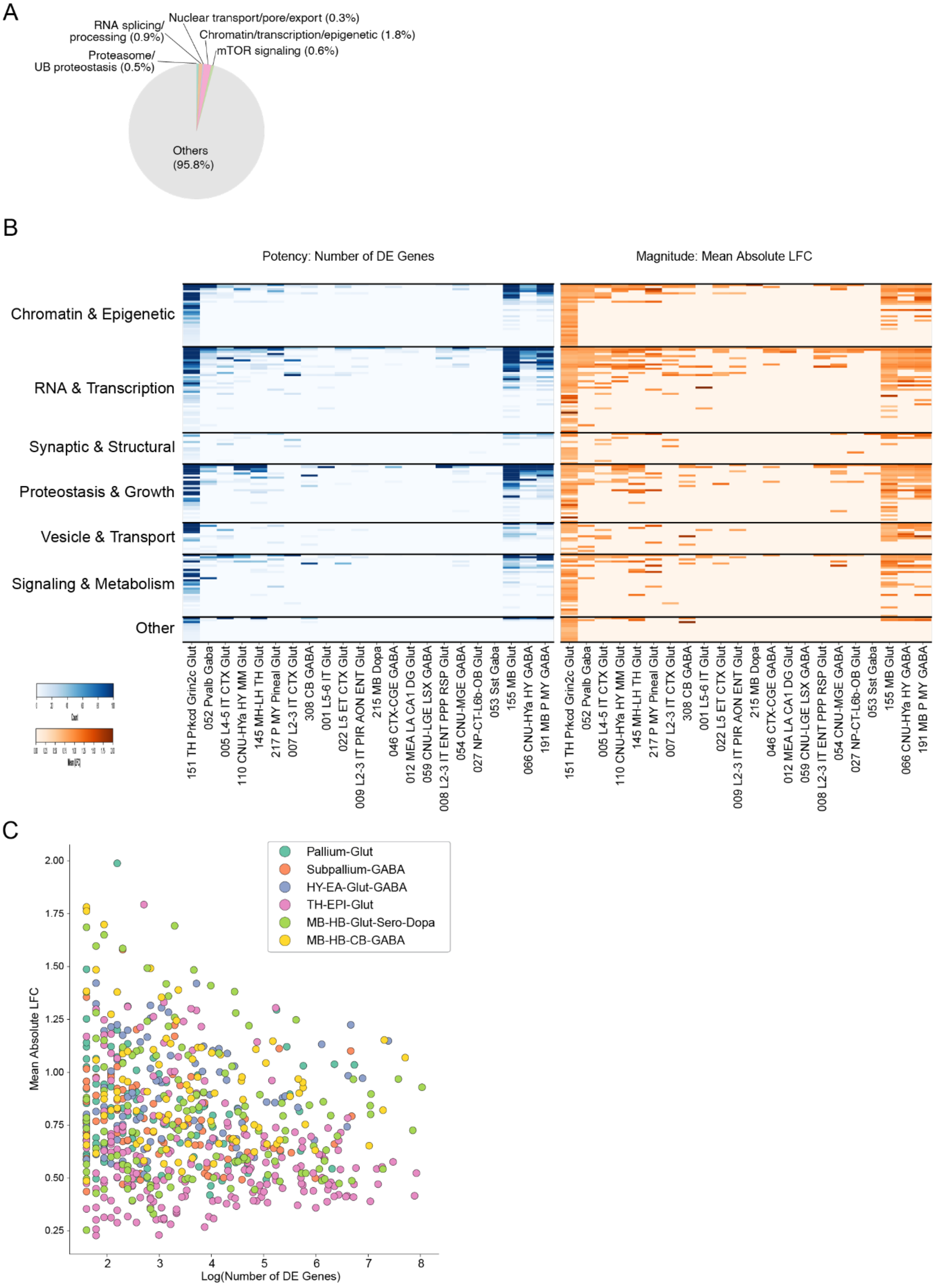

Figure S6. Pathway composition and perturbation effect structure across cell types. (A) Pie chart showing pathway category composition among all 1,947 perturbed

bioRxiv preprint doi: https://doi.org/10.64898/2026.03.16.711480; this version posted March 18, 2026. The copyright holder for this preprint (which was not certified by peer review) is the author/funder, who has granted bioRxiv a license to display the preprint in perpetuity. It is made available under aCC-BY 4.0 International license.

genes, with the vast majority (95.8%) falling outside the core pathway categories that dominate among top-DEG perturbations (compared to Fig. 2D), indicating that specific molecular systems are disproportionately represented among high-impact perturbations. **(B)** Heatmaps showing perturbation potency (number of DE genes; left, blue) and magnitude (mean absolute log-fold-change; right, orange) for each perturbation across cell types, organized by functional pathway category. Each row represents a single perturbation; columns represent binned neuronal cell types. **(C)** Scatter plot of perturbation potency (log number of DE genes) versus magnitude (mean absolute LFC) across all perturbation–cell type pairs, colored by binned neuronal class. Points in the upper-left quadrant reflect perturbations with few but large-magnitude DEGs (cell-type-restricted, strong effects), while points in the lower-right reflect perturbations with many but modest-magnitude DEGs (broad, distributed effects).

bioRxiv preprint doi: https://doi.org/10.64898/2026.03.16.711480; this version posted March 18, 2026. The copyright holder for this preprint (which was not certified by peer review) is the author/funder, who has granted bioRxiv a license to display the preprint in perpetuity. It is made available under aCC-BY 4.0 International license.

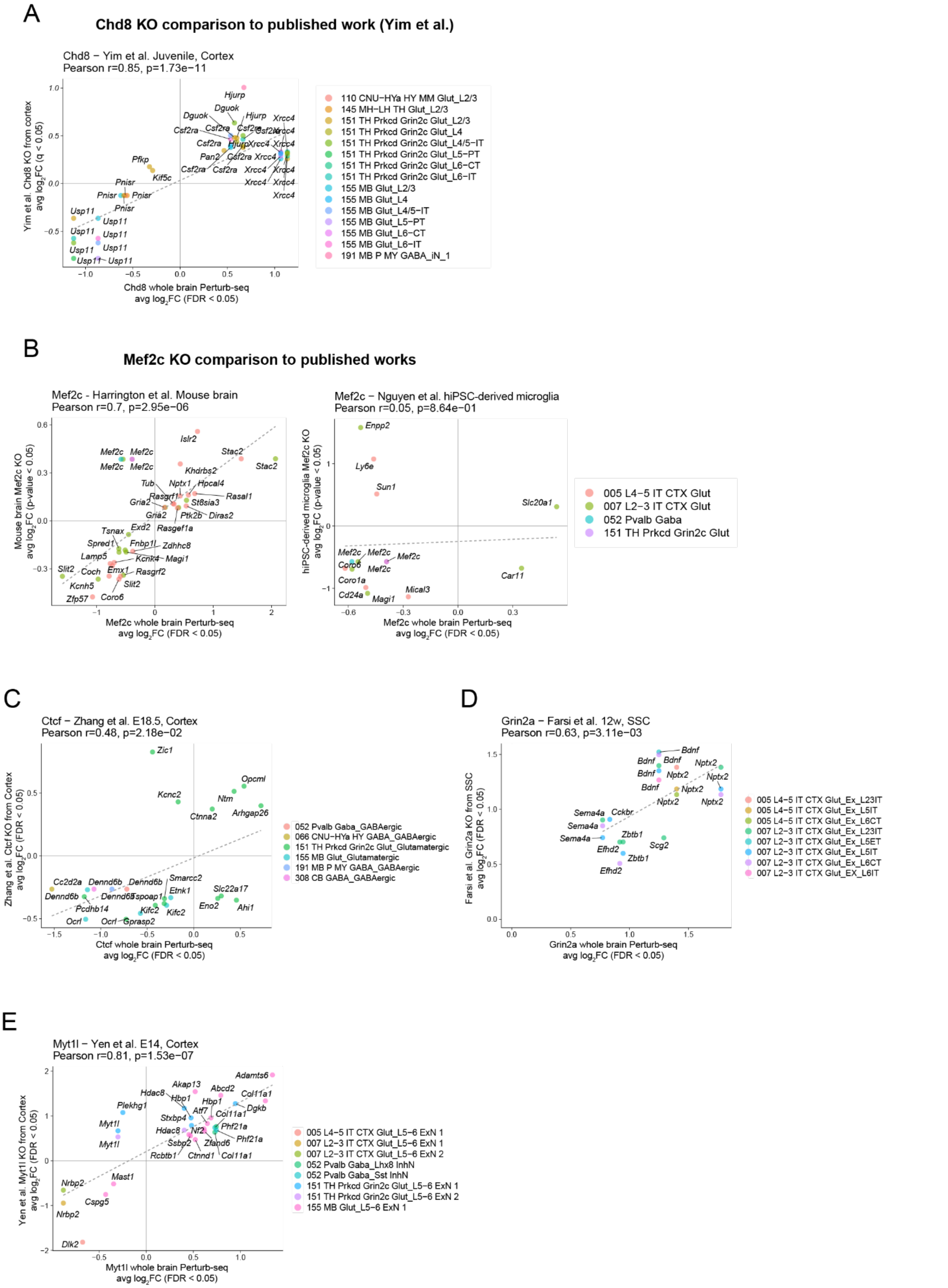

bioRxiv preprint doi: https://doi.org/10.64898/2026.03.16.711480; this version posted March 18, 2026. The copyright holder for this preprint (which was not certified by peer review) is the author/funder, who has granted bioRxiv a license to display the preprint in perpetuity. It is made available under aCC-BY 4.0 International license.

Figure S7. in vivo Perturb-seq DEG alignment with published germline or conditional knockout data. (A) *Chd8* knockout comparison with a reference *Chd8* KO dataset[16] (Yim et al., juvenile cortex). Log fold changes (logFC) of DEGs (FDR < 0.05) from the whole-brain perturb-seq dataset are compared against logFC from the reference for overlapping DEGs (q-value < 0.05). **(B)** *Mef2C* knockout comparisons with published datasets. Left: Harrington et al.[17] (mouse brain). Right: Nguyen et al.[21] (hiPSC-derived microglia). LogFC from the whole-brain perturb-seq dataset are compared against logFC from references for overlapping DEGs (p-value < 0.05). **(C-E)** Knockout comparison with reference datasets for *Ctcf* [18] (Zhang et al., E18.5 cortex) **(C)**, *Grin2a*[19] (Farsi et al. 12-week somatosensory cortex) **(D)**, *Myt1l*[20] (Yen et al., E14 cortex) **(E)**. LogFC from the whole-brain perturb-seq dataset are compared against logFC from each reference for overlapping DEGs (FDR < 0.05).

bioRxiv preprint doi: https://doi.org/10.64898/2026.03.16.711480; this version posted March 18, 2026. The copyright holder for this preprint (which was not certified by peer review) is the author/funder, who has granted bioRxiv a license to display the preprint in perpetuity. It is made available under aCC-BY 4.0 International license.

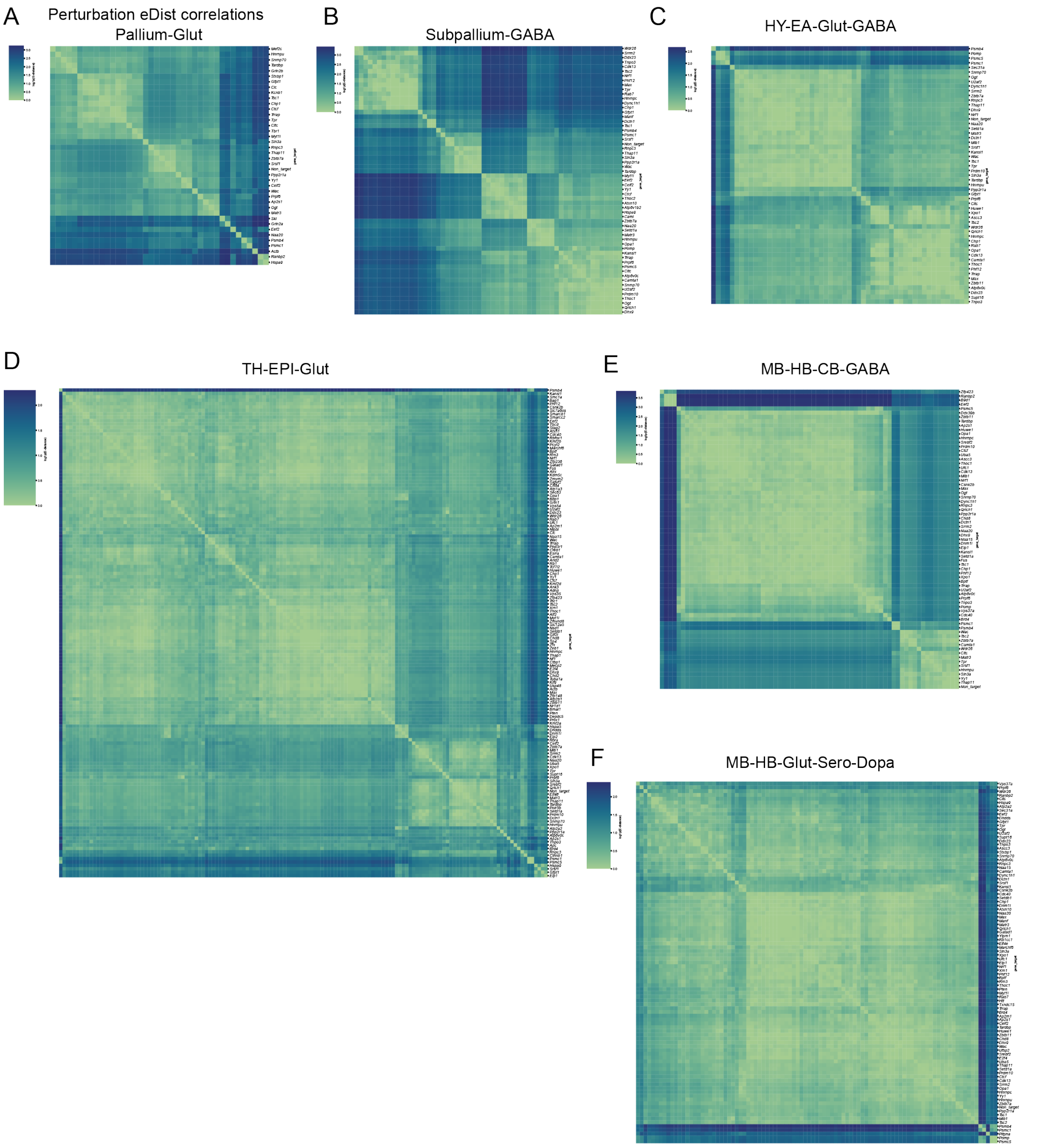

Figure S8. Cell-type-specific perturbation energy distance matrices. (A–F) Pairwise energy distance correlation matrices for effective perturbations computed within individual binned neuronal classes: Pallium-Glut (A), Subpallium-GABA (B), HY-EA-Glut-GABA (C), TH-EPI-Glut (D), MB-HB-CB-GABA (E), and MB-HB-Glut-Sero-Dopa (F). Perturbations are hierarchically clustered within each cell type. While modular structure is apparent across all classes, the specific clustering patterns differ between neuronal types, indicating that perturbation relationships are shaped by cell-type context. These cell-type-specific patterns motivated further investigation of perturbation similarity at the level of individual perturbation–cell type pairs (Fig. 3B).

bioRxiv preprint doi: https://doi.org/10.64898/2026.03.16.711480; this version posted March 18, 2026. The copyright holder for this preprint (which was not certified by peer review) is the author/funder, who has granted bioRxiv a license to display the preprint in perpetuity. It is made available under aCC-BY 4.0 International license.

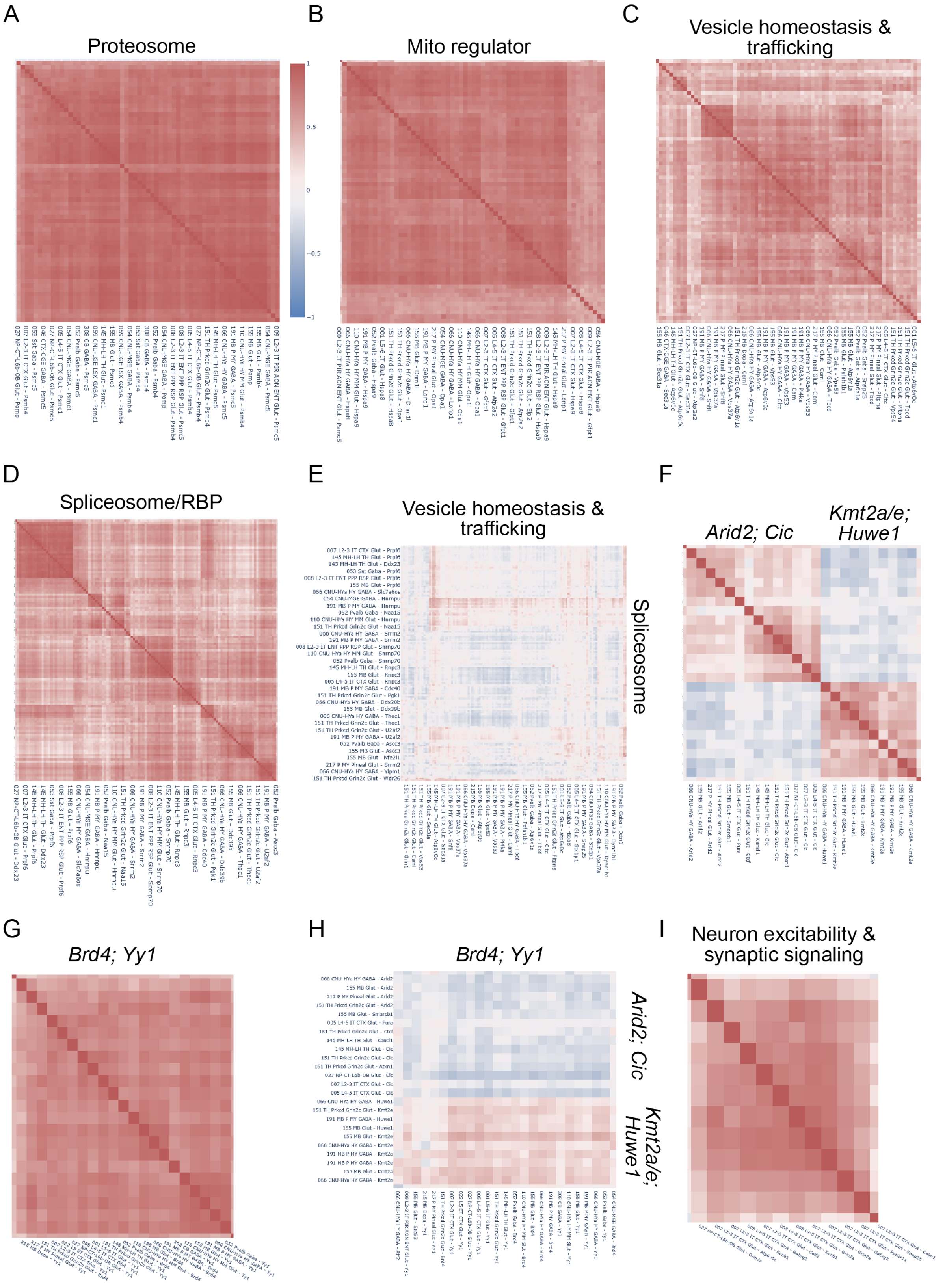

Figure S9. Cosine similarity matrices for perturbation modules identified by hierarchical clustering. (A) Pairwise cosine similarity among proteasome-related

bioRxiv preprint doi: https://doi.org/10.64898/2026.03.16.711480; this version posted March 18, 2026. The copyright holder for this preprint (which was not certified by peer review) is the author/funder, who has granted bioRxiv a license to display the preprint in perpetuity. It is made available under aCC-BY 4.0 International license.

perturbation–cell type pairs, showing high internal concordance. **(B)** Mitochondrial quality-control regulators (*Hspa5*, *Hspa8*, *Hspa9*, *Lonp1*, *Opa1*, *Dnm1l*), forming a tightly correlated module. **(C)** Vesicle homeostasis and trafficking module, including endosomal and secretory machinery components (*Vps37a*, *Vps53*, *Snf8*, *Atp6v0c*, *Atp6v1a*, *Sec31a*, *Pitpna*, *Tbcd*). **(D)** Spliceosome and RNA-binding protein module (*Prpf6*, *Snrnp70*, *Rnpc3*, *U2af2*, *Srrm2*). **(E)** Cross-module similarity between vesicle homeostasis/trafficking and spliceosome modules, showing antagonistic (negative) correlation between these functional classes. Rows and columns are labeled by perturbation–cell type pair. **(F)** Chromatin remodeling sub-modules, including SWI/SNF components and histone modifiers (*Kmt2a/e*, *Huwe1*) and a separate *Arid2*/*Cic* cluster, showing related but distinct transcriptional signatures. **(G)** *Brd4*/*Yy1*-associated transcriptional activation module. **(H)** Cross-module similarity between *Brd4*/*Yy1* and chromatin repressor modules (*Arid2*/*Cic*, *Kmt2a/e*/*Huwe1*), revealing an antagonistic axis between transcriptional activation and repressive chromatin regulators. **(I)** Neuronal excitability and synaptic signaling module (*Grin2a*, *Kcnb1*, *Gabrg2*, *Snap25*, *Celf2*, *Ppp2r1a*), with cross-module relationships to chromatin sub-modules shown.

bioRxiv preprint doi: https://doi.org/10.64898/2026.03.16.711480; this version posted March 18, 2026. The copyright holder for this preprint (which was not certified by peer review) is the author/funder, who has granted bioRxiv a license to display the preprint in perpetuity. It is made available under aCC-BY 4.0 International license.

A

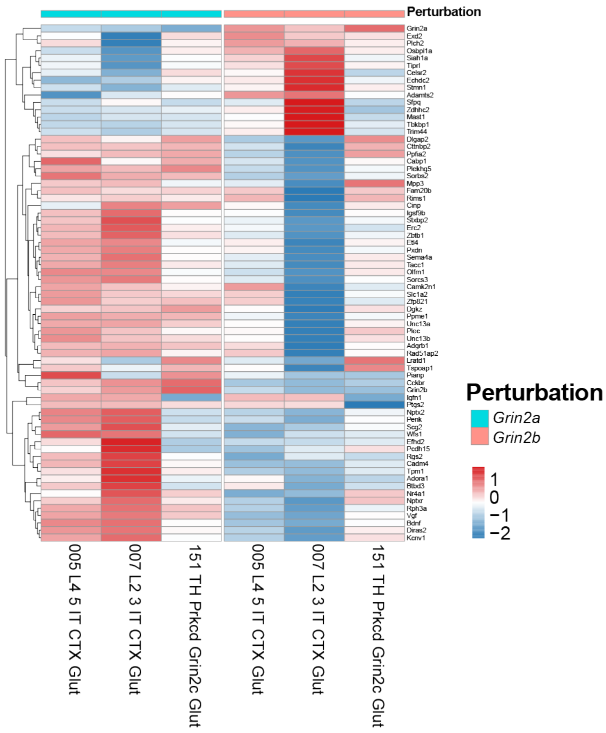

Figure S10. Context-specific perturbation effects of Grin2a and Grin2b. (A) Heatmap of selected DEGs showing variable opposing regulation between Grin2a and Grin2b perturbations in different cell types.

bioRxiv preprint doi: https://doi.org/10.64898/2026.03.16.711480; this version posted March 18, 2026. The copyright holder for this preprint (which was not certified by peer review) is the author/funder, who has granted bioRxiv a license to display the preprint in perpetuity. It is made available under aCC-BY 4.0 International license.

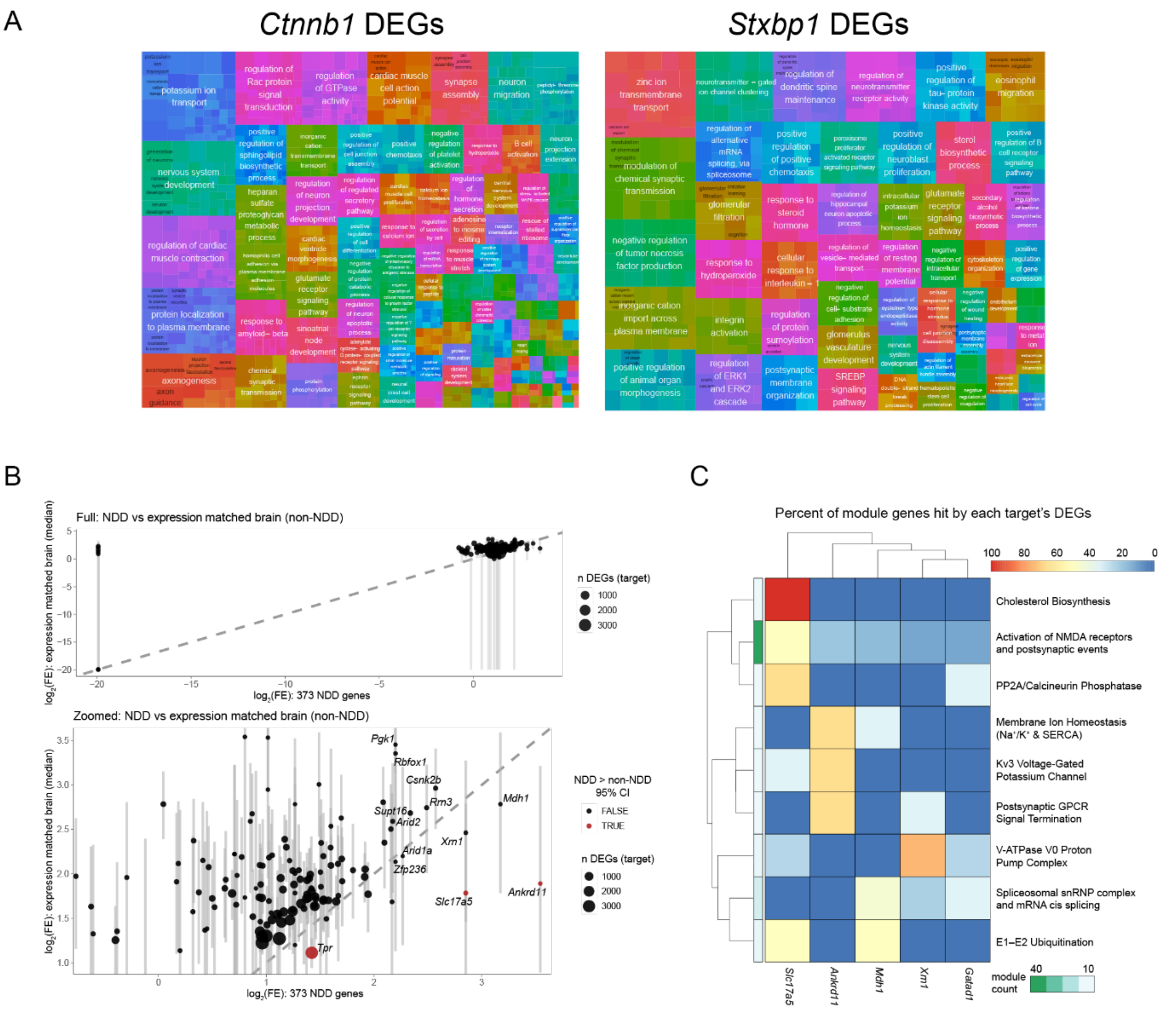

Figure S11. NDD gene enrichment and functional convergence of perturbation targets. (A) Treemap visualization of Gene Ontology Biological Process terms enriched with DEGs under perturbation of Ctnnb1 (left) and Stxbp1 (right). **(B)** Enrichment of downstream DEGs with NDD risk genes (n = 373) compared to an expression-matched brain background. Each point represents a perturbation target; point size reflects the number of DEGs. The dashed line indicates equivalence between enrichment against NDD genes and matched controls. The lower panel shows a zoomed view highlighting individual targets. Error bars represent 95% empirical confidence intervals (CI) from 1,000 bootstraps. Targets with full 95% CIs for enrichment of expression-matched genes smaller than enrichment for NDD genes are in red. **(C)** Heatmap showing percent of module genes per cluster identified in the STRING PPI network.
